# Prevalence and Clinical Characteristics of Lacunar Stroke: A Hospital-Based Study

**DOI:** 10.3390/brainsci11111466

**Published:** 2021-11-05

**Authors:** Mohammed A. Aldriweesh, Waleed A. Alluhidan, Bayan A. Al Bdah, Muath A. Alhasson, Sultan A. Alsaif, Abrar A. Alajlani, Faisal M. Almutairi, Mohammed A. Alskaini, Naser Alotaibi, Ali M. Al Khathaami

**Affiliations:** 1 College of Medicine, King Saud Bin Abdulaziz University for Health Sciences, Riyadh 14611, Saudi Arabia; almutari025@ksau-hs.edu.sa (F.M.A.); Alotaibina18@ngha.med.sa (N.A.); 2 King Abdullah International Medical Research Center, Riyadh 11481, Saudi Arabia; albdahba@ngha.med.sa; 3 College of Medicine, Imam Mohammad Ibn Saud Islamic University, Riyadh 11564, Saudi Arabia; wahalluhidan@sm.imamu.edu.sa; 4 Unaizah College of Medicine, Qassim University, Buraydah 56219, Saudi Arabia; 341103228@qu.edu.sa; 5 College of Medicine, Almaarefa University, Riyadh 13713, Saudi Arabia; 151120006@student.mcst.edu.sa (S.A.A.); 101221081@student.mcst.edu.sa (A.A.A.); 6 Department of Neurology, Prince Sultan Military Medical City, Riyadh 12233, Saudi Arabia; alskainimo@gmail.com; 7 Division of Neurology, Department of Medicine, King Abdulaziz Medical City, National Guard Health Affairs, Riyadh 11426, Saudi Arabia

**Keywords:** ischemic stroke, lacunar, cerebral small-vessel disease, prevalence

## Abstract

Lacunar stroke (LS) is responsible for one-quarter of the overall number of ischemic strokes with long-term complications and carries health and economic issues for patients and health care systems. Therefore, we aimed to investigate lacunar versus non-lacunar strokes in a tertiary academic center. From February 2016 to July 2019, all patients admitted to the stroke unit were retrospectively reviewed. We included LS patients and compared them to other TOAST subtypes. Hemorrhagic stroke and conditions mimicking stroke were excluded. Regression analysis was done to determine LS predictors and outcomes. A 35.5% rate of LS among 989 ischemic stroke patients was found. Most patients (71.9%) were males. Lower National Institutes of Health Stroke Scale (NIHSS) scores at admission and negative history for cardiac diseases were predictors for LS in our population. At discharge, LS patients had low NIHSS scores and shorter hospitalization periods compared to non-LS patients. In conclusion, LS was prevalent among ischemic stroke patients in our cohort. Future studies are highly needed with long follow-up intervals to identify the stroke recurrence, complications, and outcomes.

## 1. Introduction

Stroke is a leading cause of morbidity and mortality worldwide [1]. The absolute number of first-time ischemic strokes is increasing compared with the number of hemorrhagic strokes [2]. In Saudi Arabia, an epidemiological model predicted an increase of 67% of first-time stroke regardless of the stroke type [3]. According to the Trail of Org 10,172 in Acute Stroke Treatment (TOAST), ischemic stroke has five subtypes based on stroke etiology [4]: (1) large-artery atherosclerosis (LAA), (2) cardio-embolism (CE), (3) small-vessel occlusion “lacune” (SVO), (4) stroke of other determined etiology (OE), and (5) stroke of undetermined etiology (UE) [4]. TOAST defines SVO, lacunar stroke (LS), as “small sub-cortical brain infarcts visible on MRI (normal computed tomography scan if evaluated in the acute phase) <1.5 cm in axial diameter and associated with one of the traditional clinical lacunar syndromes” [4,5]. The prevalence of LS is about 20% among all cases of ischemic stroke [6,7,8]. In Saudi Arabia, reports addressing LS are limited. However, LS appears to be responsible for around 30% of the cases in the country [9]. Despite the high prevalence of LS, the causes and pathophysiology of this form are unclear. Cerebral small vessel disease (cSVD) is the most strongly linked to LS [5,10]. Furthermore, modifiable vascular risk factors including hypertension and type 2 diabetes mellitus (HTN and T2DM, respectively) have been associated with LS; however, their role in LS is questionable. Moreover, some reports have suggested a strong relationship between modifiable vascular risk factors and LS [11,12,13,14]. Nevertheless, some studies have applied the risk factor-free ischemic stroke subtype definitions and found no differences that could be attributed to HTN and T2DM with respect to either LS or non-LS [12]. Much of the current literature on neurocognitive diseases pays particular attention to LS as a relevant cause. In a recent systematic review and meta-analysis involving more than 2000 LS patients, the incidence of mild cognitive impairment (MCI) or post-stroke dementia was 24% [15]. Based on the criteria and exclusion of pre-stroke dementia, the incidence varied significantly between population- and hospital-based studies (7% and 41%, respectively) [16]. A study that followed ischemic stroke patients, precisely LS, for 25 years found that dementia increased twice compared with the rest of the population [17]. For Alzheimer’s disease, a 50% increase in incidence was observed after the first year [17]. The outcomes of LS differ significantly as some patients may have satisfactory recoveries, and others have a disability such as dependency, depression, and cognitive impairment [18]. Some factors such as age, male gender, second stroke, National Institutes of Health Stroke Scale (NIHSS) score upon admission, pre-stroke modified Rankin Scale (mRS), and T2DM, might predict the outcomes [17,19,20]. In the Kingdom, to our knowledge, no previous study has investigated LS patients compared to other ischemic stroke subtypes based on TOAST classification.

Therefore, we aimed to investigate LS prevalence, clinical characteristics, and outcomes and compared these parameters to other TOAST subtypes at a tertiary academic center.

## 2. Materials and Methods

### 2.1. Study Design, Area, and Settings

This retrospective cohort study was conducted at the stroke unit of King Abdulaziz Medical City, Ministry of National Guard Health Affairs, Riyadh, Saudi Arabia (KAMC-RD). KAMC-RD is a joint commission accredited tertiary academic center with more than a 1600-bed capacity. The hospital receives more than 600 stroke patients in the emergency department per year. We offer a comprehensive stroke program with a 24/7 stroke team service at our center. Almost all ischemic stroke patients undergo imaging modalities including computed tomography and CT angiography (CT and CTA, respectively) of extra and intracranial arteries. If CTA is contraindicated, the patient undergoes magnetic resonance angiography (MRA) of the neck and circle of Willis. If the initial CT brain does not show the infarct, magnetic resonance imaging (MRI) is conducted to confirm the occurrence of a stroke.

### 2.2. Study Participants

**Figure 1:**
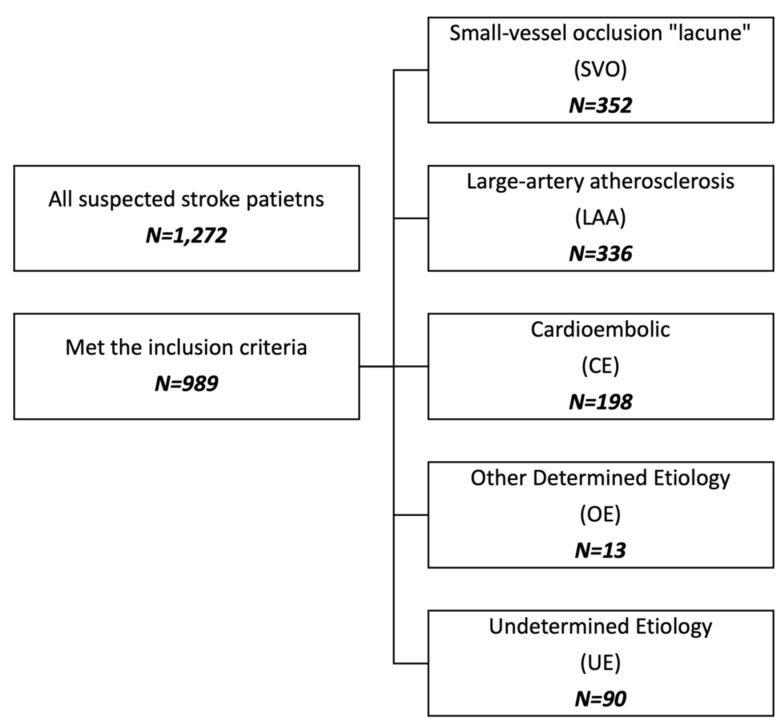
Study participants.

In this study, we included all patients who had a confirmed diagnosis of ischemic stroke and were designated to one of the five specific subtypes according to the Trail of Org 10,172 in Acute Stroke Treatment (TOAST): (1) Large-artery atherosclerosis (LAA); (2) small-vessel occlusion (SVO) “Lacunar stroke (LS)”; (3) cardioembolic (CE); (4) stroke of other determined etiology (OE); or (5) stroke of undetermined etiology (UE).) [4]. We excluded patients with hemorrhagic stroke, transient ischemic attack (TIA), cerebral sinus thrombosis (CVT), or any condition mimicking a stroke. A stroke neurologist made the final diagnosis (see Figure 1 for further information).

### 2.3. Data Collection

Data were collected from electronic health records. In addition, the following variables were collected: (1) demographics; (2) risk factors; (3) length of hospital stay; (4) modified Rankin scale (mRS) at admission and discharge; (5) NIHSS at admission and discharge; (6) final diagnosis; and (7) in-hospital death.

### 2.4. Statistical Analysis

Data were analyzed using Statistical Package for Social Sciences (SPSS) v. 22 (Chicago, IL, USA). We compared the patients who had SVO to those with LAA and CE. Data were presented as mean and standard deviation (SD) for continuous variables and frequency with percentages for categorical variables. Chi-square and/or Fisher’s exact test were used for the association between categorical variables. A *t*-test was used for normally distributed continuous variables, and Kruskal–Wallis tests were used for continuous variables based on the data. We tested for potential predictors of LS among all patients with suspected stroke using multivariable logistic regression analyses. The model included age, gender, comorbidities, stroke severity, and NIHSS score at admission. All statistical tests were considered significant at *p* < 0.05.

### 2.5. Ethical Approval

The study received approval from the Institutional Review Board (IRB) committee (RSS19/042/R) at King Abdullah International Medical Research Center (KAIMRC), the Ministry of National Guard Health Affairs.

## 3. Results

From February 2016 to July 2019, the final cohort contained 989 ischemic stroke patients (SVO: 35.5%; *N* = 352; LAA: 33.9%; *N* = 336; CE: 20%; *N* = 198; OE: 1.3%; *N* = 13; UE: 9.1%; *N* = 90). The prevalence of LS was 35.3% (*N* = 352; 95% confidence interval [CI] 32.8–38.6). Most of the LS patients were male (71.9% male; 28.1% female). The baseline characteristics of ischemic stroke based on TOAST classification are presented in Table 1. The mean age of LS patients was found to be 61 ± 11 years. Regarding vascular risk factors, 74.4% (*N* = 263) of patients with history of HTN followed by T2DM (70.5%; *N* = 248) were involved. IHD was found in 7.4% and dyslipidemia in 34.9% among LS patients. Moreover, 21.3% (*N* = 75) had a previous history of ischemic stroke or TIA. Only a few patients were smokers or had a previous smoking history (15.1%; *N* = 53). The mean mRS score at admission was 0 ± 1. Additionally, the mean NIHSS score at admission was (5 ± 4).

Regarding the hospital stay and outcomes, two LS patients died during the hospital stay. The in-hospital complications were: UTI 3.4%, pneumonia 1.1%, and only one patient developed DVT/PE. Moreover, the median NIHSS score was 2 (interquartile range [IQR} = 5), and most of the patients spent four days in the hospital (IQR 5). Based on the Barthel index for Activities of Daily Living (DALY), most patients were independent at discharge (84 ± 23). At three months, five patients (6.8%) developed a new stroke or TIA. For further information, see the table below.

Table 2 shows the comparison of LS patients and non-LS patients. A *t*-test was used to analyze the relationship between the mean age in LS and non-LS patients and found a statistical difference; *p* = 0.004, where LS patients are usually younger. Similarly, a statistical difference does exist regarding the gender between LS and non-LS with a male predominance; *p* = 0.013. Moreover, LS patients had less medical history for ischemic heart disease (IHD; 7.4%; *N* = 26; *p* ≤ 0.0001). Atrial fibrillation and dyslipidemia occurred more in the non-lacunar group [*p* ≤ 0.0001]. At admission, non-LS patients had a higher NIHSS score (5 ± 4 versus 8 ± 6 and 10 ± 7; *p* ≤ 0.0001). In-hospital complications occurred more in the non-LS group including pneumonia and urinary tract infections (*p* = ≤ 0.0001 and *p* = 0.009, respectively). More intensive care unit (ICU) admissions were observed in the non-LS patients (2% versus 12.8% and 16.4%; *p* ≤ 0.0001). The median length of hospitalization between lacunar and non-lacunar patients was compared using the Kruskal–Wallis test where a statistical difference was found, *p* ≤ 0. 0001. Regarding the outcomes, there were significant statistical differences in several categories: (1) In-hospital mortality; (2) median dependency at discharge; (3) median National Institutes of Health Stroke Scale (NIHSS) score at discharge; and (4) treatment with thrombolysis; *p* ≤ 0.0001. Moreover, the mean mRS at three months was [1 versus 3 ± 2 in both groups; no statistical difference]. The rest of the variables showed a negative statistical significance. For further information, see the table below.

Table 3 demonstrates a multivariate logistic regression of LS. The absence of ischemic heart disease (IHD), low NIHSS, and mRS score at admission, and history of ischemic stroke or TIA showed a possible association with LS in our population.

## 4. Discussion

This paper investigated the prevalence of LS, clinical characteristics, and outcomes in a single tertiary center in Riyadh, Saudi Arabia. Overall, patients with LS in our cohort had a better clinical profile when compared to the non-LS group.

Our study’s prevalence of LS was 35.5%, which is higher than the range found in other reports. In an epidemiological review, the estimated prevalence of LS ranged from 8.9–59.7% among acute ischemic stroke subtypes in the Middle East [21]. Internationally, many reports consider up to 30% of ischemic stroke as LS [6,7,22,23]. The prevalence of LS in our cohort is relatively higher than those in England (27%), Germany (25.8%), France (26.8%), Indonesia (26.7%), and Iran (22.5%) [24,25,26,27,28]. Moreover, a recent systematic review and meta-analysis over 22 years found LS to be responsible for 24% of all cases [29]. The difference in prevalence might be explained by the sample size number and cases included in our study (first-time and recurrent strokes).

T2DM and HTN have been linked to the formation of systemic and intracranial atherosclerosis leading to arterial segmentation, disorganization, and lipohyalinosis, thus increasing the intrinsic pathology of the small penetrating arteries [6,30,31,32]. In this study, 74% of the LS patients had a history of HTN; however, it was insignificant compared to those in the non-LS group. This finding is also the case in a population-based study spanning 17 years in which they found no correlation between HTN and LS [23].

Moreover, another study compared HTN as a risk factor in young and older patients with LS and found no statistical difference between the two groups [33]. However, regardless of the statistical difference concerning HTN in the previously mentioned studies and ours, HTN remains a critical vascular risk factor that needs to be controlled. T2DM was common among LS patients but had no statistical significance when compared to non-LS patients. However, T2DM and LS have been linked in previous studies [5,11,12]. Furthermore, a published study comparing the clinical profile of the ischemic stroke between patients with diabetes versus no diabetes history indicated that LS was more prevalent among patients with diabetes [13]. Moreover, LS in diabetic patients shows a poor patient prognosis with respect to recurrent stroke and death [34]. 

Finally, our study had some limitations including the cohort nature of the study, in which some cases might have been overlooked. Moreover, we did not follow the patients at different time intervals to estimate and identify the predictors of recurrent stroke and calculate the mortality rate for more than three months. Additionally, the results cannot be generalized since it was a single-center study. On the other hand, to the best of our knowledge, this is the first study to describe LS in-depth in a Saudi population, and the study had a good sample of patients.

## 5. Conclusions

In conclusion, we investigated 989 ischemic stroke patients for LS prevalence, clinical characteristics, and outcomes. LS accounted for 35.5% of all cases in our study. Many patients had a moderate stroke based on the NIHSS score upon admission. LS patients tended to have better clinical profiles than non-LS patients before the onset of stroke. Patients with LS had a favorable in-hospital stay and improvement in the NIHSS at discharge. Finally, LS is burdensome and prevalent among ischemic stroke patients in Saudi Arabia and needs to be further studied to determine its outcomes over a long-term period with a larger population.

## Figures and Tables

**Table 1:** Baseline characteristics of the patients (*N* = 989) with ischemic stroke based on TOAST classification.

	Small-Vessel Occlusion (SVO) *“LS”* *N* = 352	Large-Artery Atherosclerosis (LAA) *N* = 336	Cardioembolic (CE) *N* = 198	Other Determined Etiology (OE) *N* = 13	Undetermined Etiology (UE) *N* = 90
Characteristics	*N* (%)				
Mean age (years) ± standard deviation (SD)	61 ± 11	61 ± 12	64 ± 12	47 ± 18	55 ± 14
Gender					
- Male	253 (71.9)	226 (67.3)	118 (59.6)	8 (61.5)	67 (74.4)
- Female	99 (28.1)	110 (32.7)	80 (40.4)	5 (38.5)	23 (25.6)
Medical history					
- Hypertension	263 (74.7)	238 (70.8)	147 (74.2)	7 (53.8)	53 (58.9)
- Diabetes mellitus	248 (70.5)	222 (66.1)	117 (59.1)	8 (61.5)	48 (53.3)
- Dyslipidemia	123 (34.9)	97 (28.9)	54 (27.3)	2 (15.4)	25 (27.8)
- Ischemic heart disease	26 (7.4)	40 (11.9)	37 (18.7)	0	7 (7.8)
- Atrial fibrillation	7 (2)	7 (2.1)	64 (32.3)	0	1 (1.1)
- Valvular heart disease	6 (1.7)	4 (1.2)	21 (10.6)	0	2 (2.2)
Smoker/history of smoking	53 (15.1)	55 (16.4)	34 (17.2)	1 (7.7)	26 (28.9)
History of ischemic stroke or TIA	75 (21.3)	90 (26.8)	55 (27.8)	5 (38.5)	15 (16.7)
Mean modified Rankin Scale at admission ± SD (mRS)	0 ± 1	1 ± 1	1 ± 1	1 ± 1	0 ± 1
Mean NIHSS score at admission ± SD	5 ± 4	8 ± 6	10 ± 7	7 ± 4	6 ± 6
In-hospital Complications					
Pneumonia	4 (1.1)	25 (7.4)	14 (7.1)	0	3 (3.3)
Urinary tract infection	12 (3.4)	29 (8.6)	17 (8.6)	2 (15.4)	1 (1.1)
Deep vein thrombosis/Pulmonary embolism	1 (0.3)	5 (1.5)	5 (2.5)	1 (7.7)	0
Intensive care unit (ICU) admissions	7 (2)	43 (12.8)	32 (16.4)	3 (23.1)	10 (11.1)
The median length of stay (IQR) (Days)	4 (5)	8 (13)	10 (18)	17 (18)	4 (6)
Outcome					
In-hospital mortality	2 (0.6)	16 (4.8)	13 (6.6)	1 (7.7)	0
Mean Dependency at discharge ± SD	84 ± 23	63 ± 35	60 ± 36	69 ± 41	81 ± 20
Median NIHSS at discharge (IQR)	2 (5)	4 (7)	5 (10)	1 (6)	2 (4)
Mean modified Rankin Scale at discharge ± SD (mRS)	1 ± 1	2 ± 2	3 ± 2	2 ± 2	1 ± 2
Treatment with Tissue plasminogen activator (tPA)	15 (4.3)	52 (15.5)	33 (16.7)	1 (7.7)	4 (4.4)
Endovascular thrombectomy (EVT)	0	26 (7.7)	19 (9.6)	1 (7.7)	4 (4.4)
Recurrent stroke/TIA at three months	5 (6.8)	11 (11.1)	3 (6.1)	0	2 (9.1)
Mean modified Rankin Scale at three months ± SD (mRS)	1 ± 1	3 ± 2	3 ± 2	NA	1 ± 0

**Table 2:** A comparison of LS patients and other ischemic stroke based on TOAST classification.

	Small-Vessel Occlusion (SVO) *“LS”* *N* = 352	Large-Artery Atherosclerosis (LAA) *N* = 336	Cardioembolic (CE) *N* = 198	*p*
Characteristics	*N* (%)			
Mean age (years) ± standard deviation (SD)	61 ± 11	61 ± 12	64 ± 12	0.004
Gender				
- Male	253 (71.9)	226 (67.3)	118 (59.6)	0.013
- Female	99 (28.1)	110 (32.7)	80 (40.4)	
Medical history				
- Hypertension	263 (74.7)	238 (70.8)	147 (74.2)	0.4
- Diabetes mellitus	248 (70.5)	222 (66.1)	117 (59.1)	0.026
- Dyslipidemia	123 (34.9)	97 (28.9)	54 (27.3)	0.1
- Ischemic heart disease	26 (7.4)	40 (11.9)	37 (18.7)	<0.0001
- Atrial fibrillation	7 (2)	7 (2.1)	64 (32.3)	<0.0001
- Valvular heart disease	6 (1.7)	4 (1.2)	21 (10.6)	0.0001
Smoker/history of smoking	53 (15.1)	55 (16.4)	34 (17.2)	0.7
History of ischemic stroke or TIA	75 (21.3)	90 (26.8)	55 (27.8)	0.1
Mean modified Rankin Scale at admission ± SD (mRS)	0 ± 1	1 ± 1	1 ± 1	0.03
Mean NIHSS score at admission ± SD	5 ± 4	8 ± 6	10 ± 7	<0.0001
In-hospital Complications				
Pneumonia	4 (1.1)	25 (7.4)	14 (7.1)	<0.0001
Urinary tract infection	12 (3.4)	29 (8.6)	17 (8.6)	0.009
Deep vein thrombosis/Pulmonary embolism	1 (0.3)	5 (1.5)	5 (2.5)	0.03
Intensive care unit (ICU) admissions	7 (2)	43 (12.8)	32 (16.4)	<0.0001
The median length of stay (IQR) (Days)	4 (5)	8 (13)	10 (18)	<0.0001
Outcome				
In-hospital mortality	2 (0.6)	16 (4.8)	13 (6.6)	<0.0001
Mean Dependency at discharge ± SD	84 ± 23	63 ± 35	60 ± 36	<0.0001
Median NIHSS at discharge (IQR)	2 (5)	4 (7)	5 (10)	<0.0001
Mean modified Rankin Scale at discharge ± SD (mRS)	1 ± 1	2 ± 2	3 ± 2	<0.0001
Treatment with Tissue plasminogen activator (tPA)	15 (4.3)	52 (15.5)	33 (16.7)	<0.0001
Endovascular thrombectomy (EVT)	0	26 (7.7)	19 (9.6)	<0.0001
Recurrent stroke/TIA at three months	5 (6.8)	11 (11.1)	3 (6.1)	0.4
Mean modified Rankin Scale at three months ± SD (mRS)	1 ± 1	3 ± 2	3 ± 2	0.1

**Table 3:** Multivariate logistic regression analysis of small-vessel occlusion (SVO) “(LS)” in ischemic stroke patients.

Independent Variable	Adjusted Odd Ratio (AOR)	95% CI for OR		*p*
		Lower	Upper	
Age	0.998	0.984	1.012	0.7
Male gender Female (Reference)	1.3	0.903	1.872	0.1
Medical history:				
Ischemic heart disease	2.128	1.218	3.716	0.008
Diabetes mellitus	0.822	0.561	1.205	0.3
Dyslipidemia	0.875	0.609	1.257	0.4
Atrial fibrillation	0.982	0.327	2.948	0.9
Valvular heart disease	0.816	0.219	3.046	0.7
History of ischemic stroke or TIA	1.522	1.042	2.223	0.03
NIHSS score at admission	0.888	0.859	0.918	<0.0001
The dependent outcome is dichotomous (SVO “LS” versus LAA) The reference group is LAA				

## Data Availability

The data presented in this study are available on request from the corresponding author. The data are not publicly available as per the KAIMRC ethics committee policy, the data of this study are not approved to be released publicly, and the data supporting the findings of this study will be available upon reasonable request. Data requests can be sent to the corresponding author or King Abdullah International Medical Research Center, mailing address P.O. Box 3660, Riyadh 11481, Mail Code 1515 (KAIMRC), Tel.: +96-6429-4444, Email: KAIMRC@NGHA.MED.SA.
